# Child abuse inventory at emergency rooms: CHAIN-ER rationale and design

**DOI:** 10.1186/1471-2431-11-91

**Published:** 2011-10-18

**Authors:** Judith S Sittig, Cuno SPM Uiterwaal, Karel GM Moons, Edward ES Nieuwenhuis, Elise M van de Putte

**Affiliations:** 1 General Pediatrics, Wilhelmina Children's Hospital, University Medical Centre Utrecht, The Netherlands; 2 Julius Center for Health Sciences and Primary Care, University Medical Centre Utrecht, The Netherlands

## Abstract

**Background:**

Child abuse and neglect is an important international health problem with unacceptable levels of morbidity and mortality. Although maltreatment as a cause of injury is estimated to be only 1% or less of the injured children attending the emergency room, the consequences of both missed child abuse cases and wrong suspicions are substantial. Therefore, the accuracy of ongoing detection at emergency rooms by health care professionals is highly important. Internationally, several diagnostic instruments or strategies for child abuse detection are used at emergency rooms, but their diagnostic value is still unknown. The aim of the study 'Child Abuse Inventory at Emergency Rooms' (CHAIN-ER) is to assess if active structured inquiry by emergency room staff can accurately detect physical maltreatment in children presenting at emergency rooms with physical injury.

**Methods/design:**

CHAIN-ER is a multi-centre, cross-sectional study with 6 months diagnostic follow-up. Five thousand children aged 0-7 presenting with injury at an emergency room will be included. The index test - the SPUTOVAMO-R questionnaire- is to be tested for its diagnostic value against the decision of an expert panel. All SPUTOVAMO-R positives and a 15% random sample of the SPUTOVAMO-R negatives will undergo the same systematic diagnostic work up, which consists of an adequate history being taken by a pediatrician, inquiry with other health care providers by structured questionnaires in order to obtain child abuse predictors, and by additional follow-up information. Eventually, an expert panel (reference test) determines the *true *presence or absence of child abuse.

**Discussion:**

CHAIN-ER will determine both positive and negative predictive value of a child abuse detection instrument used in the emergency room. We mention a benefit of the use of an expert panel and of the use of complete data. Conducting a diagnostic accuracy study on a child abuse detection instrument is also accompanied by scientific hurdles, such as the lack of an accepted reference standard and potential (non-) response. Notwithstanding these scientific challenges, CHAIN-ER will provide accurate data on the predictive value of SPUTOVAMO-R.

## Background

The World Health Organization has recognized child abuse and neglect as a major international health problem [1] with unacceptable levels of morbidity and mortality [2]. Child maltreatment encompasses any acts of commission or omission by a parent or other caregiver that result in harm, potential for harm, or threat of harm to a child, even if harm is not the intended result [3]. Four forms of maltreatment are widely recognized: physical abuse, sexual abuse, neglect and emotional abuse. Increasingly, witnessing intimate-partner violence is also regarded as a separate form of child maltreatment [4]. In high-income countries, the annual incidence of self-/parent- reported physical abuse is 4-16%, the annual incidence of neglect is 1.4-15.4% and the annual incidence of psychological abuse is 10.3% [4]. Although maltreatment as a cause of injury is estimated to be only 1% or less of the injured children attending the emergency room (ER) [5], the consequences of both missed child abuse cases and wrong suspicions are substantial. Missed diagnosis may have enormous influence on education, mental health, physical health, and violence or criminal behavior [6]. Besides, inaccurate suspicions also have a huge impact on children and their families. Generally the children with injury caused by child maltreatment that are seen in the ER are the most serious cases of abuse or neglect, thus putting these children at greater risk of subsequent severe maltreatment related injury or death. From a public health perspective, early identification of maltreatment allows children and families to receive intervention to prevent further maltreatment, thus reducing the cost of maltreatment to the individual, the family and the society. As stated by the American Academy of Pediatrics: 'accurate and timely diagnosis of children who are suspected victims of abuse can ensure appropriate evaluation, investigation, and outcomes for these children and their families' [7]. For all these reasons, accurate detection of child maltreatment at ER's by health care professionals is highly important.

**Figure 1:**
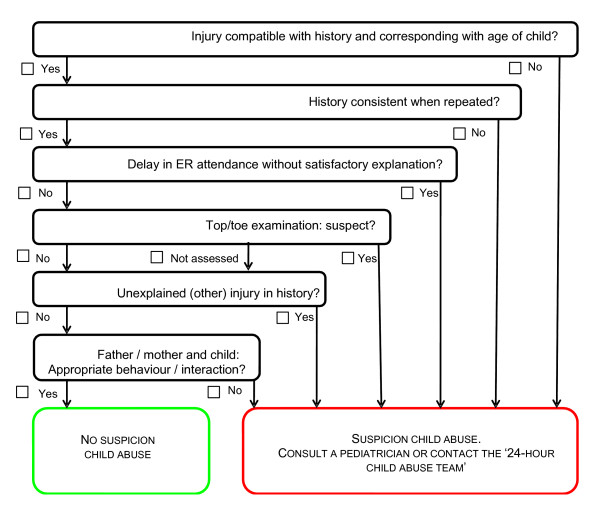
**SPUTOVAMO-R**.

Internationally, several diagnostic instruments or strategies for child abuse detection are used at ER's [8-14], such as checklists, protocols and scoring systems, sometimes restricted to particular characteristics e.g. age, type of injury, repeated attendance, or a medical history inconsistent with the injury [5,15]. For example, in the UK the checklist of Benger et al. [8] is regularly used. Four questions regarding the injury aim to lead to a distinction between physical child abuse suspicion and non-suspicion. In the Netherlands, a child abuse detection instrument called SPUTOVAMO has been widely introduced at ER's. SPUTOVAMO is an acronym composed of the first letters of 9 questions regarding the injury. This checklist, originally developed by Compernolle [16] in 1996, was revised into a checklist with 6 questions with binary answer possibilities pointing unambiguously at the suspicion of child abuse or not. This SPUTOVAMO-R (*see *figure 1) is quite similar to the detection instrument of Benger et al. [8].

The recently published meta-analysis by Woodman et al. [5] showed that there was clear evidence that physically abused children attending ER's are missed, although estimates ranged substantially (11-64%). However, the validity and applicability of the three included studies was poor. All three studies met only 2 of the 12 quality criteria listed in the Quality Assessment of Diagnostic Accuracy Studies (QUADAS) tool, an evidence based quality assessment tool for systematic reviews of diagnostic accuracy studies [17].

The conclusion of Woodman et al. [5] is in line with the conclusion of another recently published systematic review of Louwers et al. [18]. Both reviews stated the conclusion that well-designed, large-scale studies are required to validly evaluate the accuracy and effectiveness of assessments that are currently used in ER's for identifying abused children and for initiating appropriate interventions [5,18].

The study 'Child Abuse Inventory at Emergency Rooms' (CHAIN-ER) is designed to provide data in children presenting with physical injury at ER's on the predictive value of SPUTOVAMO-R in establishing a diagnosis of child abuse.

## Methods/design

### Study design

**Figure 2:**
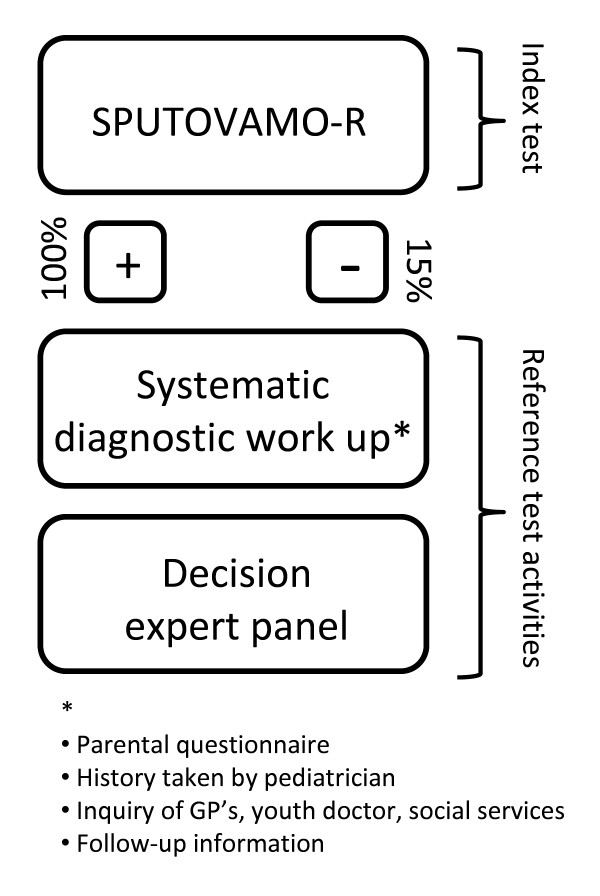
**Design of CHAIN-ER**.

A cross-sectional multi-centre study with 6 months follow-up will be conducted to assess if active structured inquiry by ER staff, using the SPUTOVAMO-R, can accurately detect physical maltreatment in children presenting at ER's with physical injury. The diagnostic value of SPUTOVAMO-R (index test) is to be tested against the consented opinion of an expert panel (reference test) (*see *figure 2).

### Study population

The study population will include 5,000 children aged 0-7 years, referred as from June 2009 to an ER because of any physical injury. Four hospitals, of which one academic and three non-academic hospitals in the region of Utrecht, a city in the centre of the Netherlands, will participate. Children injured due to witnessed traffic accidents and children who were dead at ER arrival, will be excluded. All participating ER's combine adult and pediatric care. The annual childhood (0-18 year) attendance rate of the 4 ER's varies between 4000 and 8200 visits per year. In all participating hospitals SPUTOVAMO-R is filled out for all patients under the age of 18 years attending the ER. This is a compulsory field in the electronic file of the medical records of patients under the age of 18 years.

### Procedure

In the standard clinical care, the first step in the process of recognition of child abuse is completing the SPUTOVAMO-R by ER professionals during the ER visit. SPUTOVAMO-R is to be considered positive for the suspicion of physical child abuse when at least one of the six questions is deviant. The result of this first step will predominantly determine the consecutive clinical work-up for the potentially maltreated child. A suspicion of child abuse by the ER professional (positively screened SPUTOVAMO-R) is followed by a systematic workup for possible child abuse starting with a pediatric consultation in the ER. After the work-up, all screened positive cases are discussed in the multidisciplinary Child Abuse Team in the presence of the Child Protection Services.

The research procedure for the study subjects runs parallel to the clinical work up and is as follows. All SPUTOVAMO-R positives and a random sample of the SPUTOVAMO-R negatives will undergo the same systematic diagnostic work up. For the expert panel it is unfeasible to perform such an extensive reference test in all 5,000 cases and therefore a random sample of 15% of the negative SPUTOVAMO-R cases will be taken. Before the start of the study, all ER personnel will be adequately trained [19] in objectively recognizing child abuse and in filling in the SPUTOVAMO-R form.

The diagnostic work up for this study will be supplementary to the essential clinical care and consists of several extra steps. First, pediatricians will clarify the initial injury for all study subjects. In order to be sure that the expert panel will have access to all necessary information to judge the SPUTOVAMO-R results, pediatricians will phone all included families to ask any additional questions related to the injury, which are not apparently asked or documented in the ER report. Second, identification of child abuse risk factors will be assessed by parental, general practitioners' and youth health care giver's questionnaires. These risk factors will include factors regarding the child, its parents and its family. Third, additional information about the event under study, as well as information about potential later ER visits, during a 6 months follow up period will be obtained.

All information acquired will be presented in a highly structured way to the members of the expert panel for each included case subjected to the reference test. A single reference test for the ultimate diagnosis of child abuse is obviously lacking. In CHAIN-ER the *true *presence or absence of child abuse for all the subjects under study will be determined using a consensus procedure of an expert panel consisting of three (forensic) pediatricians. All panel members are extensively trained in recognizing child abuse and work as experts in the field. The expert panel will have all subject information of the entire work up at its disposal. Well-informed and blinded to opinions of previous health care providers about individual cases, the panel will decide firstly on the nature of the injury (intentional or non-intentional), secondly on the probability that this child is victim of child abuse in a broad sense (i.e. the four types of child abuse) and thirdly on the need for help from social services in this family. Consensus is achieved when all panel members unanimously decide on the intentional or accidental nature of the injury. For the decisions about the probability of child abuse in general and the need for help from social services consensus is determined by the majority of the three expert opinions.

### Ethical approval

This study has been reviewed and approved by the Medical Ethical Committee of the University Medical Centre Utrecht (reference 08-378). At ER presentation, written information on the current research will be provided to all parents. Since we expect parents of SPUTOVAMO-R negatives to participate more easily than parents of SPUTOVAMO-R positives (so called response bias), we were willing to make the threshold of participating as low as possible for all parents. We expect that the threshold to participate will be lower when parents only have to give their oral agreement. Especially for the conduct of the CHAIN-ER project, we received permission of the Ministry of Justice (Privacy Helpdesk) that oral informed consent for the exchange of information with other health care professionals will suffice in stead of the customary written informed consent. This agreement was stated in a covenant signed by all representative organizations of the participating health care professionals. The Medical Ethical Committee of the University Medical Centre Utrecht approved this agreement as well.

### Statistical Analyses and Power Calculation

The study population will include approximately 5,000 children, of which we expect approximately 100 cases to be positive (2%). Of all SPUTOVAMO-R negatives a random sample of 15% will be taken. This will give the expert panel a work load of approximately 750 cases (650 SPUTOVAMO-R negatives and 100 SPUTOVAMO-R positives). A potential disadvantage of taking a random sample is that false negative cases can not be accurately determined when the prevalence of child abuse in cases with negative test results is very small. However, even when the prevalence of false negative test results is only 1%, a random sample of 15% will detect several (i.e. approximately 8) false negative cases.

To make it possible to determine both positive and negative predictive value (PPV and NPV) as well as the test's sensitivity and specificity of SPUTOVAMO-R, we need the panel to receive both positive and negative SPUTOVAMO-R cases. To calculate the PPV, the sampling fraction of the test negatives must be known [20], which we know in this case (1/0.15). Additional techniques that will be used to determine the value of SPUTOVAMO-R are tests of discrimination with calculation of Areas under Receiver Operating Characteristic curves, and calibration techniques to evaluate if predicted abuse corresponds to observed abuse.

To determine associations between potential child abuse risk factors and true child abuse, logistic regression is used with the opinion of the expert panel (child abuse yes/no) as dependent variable and the several risk factors as measured by questionnaires as independent variables. We expect about 100 cases (i.e. child abuse confirmed by the reference panel). For every 10 confirmed cases we could examine 1 independent variable (according to the 1:10 rule of Harrell [21]). With approximately 100 confirmed child abuse cases, we will have the opportunity to determine 10 variables. If we would determine the associations between more than 10 potential child abuse risk factors and true child abuse, we will need to reduce the number of predictors by cluster analysis. The (restricted) set of predictors will be used in the univariable logistic regression analysis. Relevant factors will be used in a multivariable logistic regression model.

## Discussion

To the best of our knowledge, CHAIN-ER will be the first study to determine both positive and negative predictive value of a child abuse detection instrument used in the ER by performing the index test and subsequently the same reference method for all study subjects. This allows us to determine both PPV and NPV of the index test under study, as well as its sensitivity and specificity. Most existing evidence on diagnostic tests for child abuse at ER's is flawed because those detected as negative are not subjected to the same reference standard as the positive detected cases.

Furthermore, we think a main advantage of the use of an expert panel is that, contrary to members of a multidisciplinary team (commonly the reference test in clinical practice), the members of the expert panel are not involved in case management of the included patients and might feel loyalty or disloyalty to the patient and its caregivers. Another advantage is that panel members, again contrary to members of a multidisciplinary team, are blinded for the result of SPUTOVAMO-R (the index test of main interest) which avoids so-called incorporation bias that could result from the test under study being incorporated in the assessment of the final diagnosis [22].

A third strength of this study is the large number of included patients. The SPUTOVAMO-R results of approximately 750 cases which are to be tested against the opinion of the reference panel will reflect a study population of approximately 5,000 children (see statistical analysis). These 5,000 children will come from both academic and non-academic hospitals. Both citizens from the city center and from rural areas will visit these hospitals, which seems to make the study population a correct reflection of the general Dutch childhood population.

However, conducting a study on diagnostic accuracy of a child abuse detection instrument is accompanied by several scientific hurdles, such as the lack of an accepted reference standard. In CHAIN-ER we will try to optimize the gold standard test by the use of the aforementioned expert panel.

In addition, differential (non-) response is a potential scientific hurdle as well. To obtain and to use medical information of the infant, informed consent of its parents is needed. It may well be that parents who maltreat their children refuse to participate. On the other hand, one may reason that in case of true child abuse, parents might in fact agree to participate in research, to avoid any suspicion of maltreatment. Nevertheless, in both ways, a certain level of differential (non-) response is inevitable. CHAIN-ER will aim to quantify the differential (non-) response concern, by checking afterwards whether a certain level of differential (non-) response may be present in the data. When parents do not want us to obtain additional information from other health care professionals, the yet available information of the ER visit, including medical history, physical examination and potential supplemental diagnostic investigations, such as the multidisciplinary team assessment are still available. Accordingly, one (e.g. an expert panel) can judge afterwards the (incomplete) case on the yet available information of the ER visit. Although such judgement will obviously be based on less information than in other subjects, investigators may still be able to decide on the (non) inflicted origin of the injury. Based on the thus obtained 'endpoints or panel decisions' investigators can determine whether there is a difference between the refusers and the non-refusers in terms of all available or observed child and injury characteristics. Accordingly, the potential of non-differential response can be as good as possible addressed by the data, and -if present- its influence on the observed accuracy of the diagnostic index tests discussed.

In conclusion, notwithstanding the scientific challenges of conducting a diagnostic accuracy study on a child abuse detection instrument, CHAIN-ER will provide accurate data on the predictive value of SPUTOVAMO-R.

## Abbreviations

CHAIN-ER: Child Abuse Inventory at Emergency Rooms; ER: emergency room; GP: general practitioners; NPV: negative predictive value; PPV: positive predictive value; QUADAS: Quality Assessment of Diagnostic Accuracy Studies; SPUTOVAMO-R: Acronym consisting of the first letters of the questions (in Dutch):

**S**oort letsel/klacht: compatibel met verhaal en passend bij leeftijd kind?

**P**ersisterend hetzelfde verhaal?

**U**itstel in hulpzoeken zonder bevredigende verklaring?

**T**op/teen onderzoek verdacht?

**O**nverklaard (ander) letsel/klacht in VG?

**Va**der/**mo**eder en kind: adequaat gedrag?

**R**evised

(For English translation: see figure 1)

## Competing interests

None of the authors has any potential financial conflict of interest related to this manuscript.

All authors declare that (1) JSS, CSPMU, KGMM, EESN and EMP have support from the Netherlands Institution for Health Research and Development (ZonMw) for the submitted work; (2) JSS, CSPMU, KGMM, EESN and EMP have no relationships with the Netherlands Institution for Health Research and Development (ZonMw) that might have an interest in the submitted work in the previous 5 years; (3) their spouses, partners, or children have no financial relationships that may be relevant to the submitted work; and (4) JSS, CSPMU, KGMM, EESN and EMP have no non-financial interests that may be relevant to the submitted work.

## Authors' contributions

JSS is primary investigator and responsible for data collection and analysis and for drafting the manuscript. CSPMU, KGMM, EESN and EMP designed and supervised the study. EMP obtained funding for the study. All authors have read and approved the final manuscript.

## Pre-publication history

The pre-publication history for this paper can be accessed here:

http://www.biomedcentral.com/1471-2431/11/91/prepub
